# Influence of Paleolithic diet on anthropometric markers in chronic diseases: systematic review and meta-analysis

**DOI:** 10.1186/s12937-019-0457-z

**Published:** 2019-07-23

**Authors:** Ehrika Vanessa Almeida de Menezes, Helena Alves de Carvalho Sampaio, Antônio Augusto Ferreira Carioca, Nara Andrade Parente, Filipe Oliveira Brito, Thereza Maria Magalhães Moreira, Ana Célia Caetano de Souza, Soraia Pinheiro Machado Arruda

**Affiliations:** 1 Postgraduate Program in Colletive Health, Universidade Estadual do Ceará (UECE), Fortaleza, Brazil; 2 Nutrition course, Universidade de Fortaleza (UNIFOR), Fortaleza, Brazil; 3 Nutrition in Public Health, Faculdade Saúde Pública, Universidade de São Paulo (USP), São Paulo, Brazil; 4 Public Health, Universidade de São Paulo (USP), São Paulo, Brazil; 5 Clinical Care in Health, Universidade Federal do Ceará (UFC), Fortaleza, Brazil; 6 Nucleus of Research and Development of Medications, Universidade Federal do Ceará, Fortaleza, Brazil; 7 Collective Health, Universidade Federal do Maranhão, São Luís, Brazil; 8 Postgraduate Program in Collective Health and Academic Master’s in Nutrition and Health, Universidade Estadual do Ceará, Fortaleza, Brazil

**Keywords:** Paleolithic diet, Anthropometry, Obesity, Systematic review, Meta-analysis

## Abstract

**Background:**

The Paleolithic diet has been studied in the scope of prevention and control of chronic noncommunicable diseases (CNCD). The objective of this study was to analyze the influence of the Paleolithic diet on the prevention and control of CNCD in humans, specifically on anthropometric markers, through a systematic review with meta-analysis.

**Methods:**

What is the effect of the Paleolithic diet on anthropometric parameters (weight, body mass index and waist circumference) compared to other control diets based on recommendations in adults? We included only randomized studies with humans that used the Paleolithic Diet in the prevention and control of CNCD published in Portuguese, English or Spanish. The search period was until March 2019, in the LILACS, PubMed, Scielo, Science Direct, Medline, Web of Science and Scopus databases. The abstracts were evaluated by two researchers. We found 1224 articles, of which 24 were selected and 11 were included in the meta-analysis. The effect of dietary use on body weight, body mass index and waist circumference was evaluated.

**Results:**

The summary of the effect showed a loss of − 3.52 kg in the mean weight (CI 95%: − 5.26; − 1.79; *p* < 0,001; I^2^ = 24%) of people who adopted the Paleolithic diet compared to diets based on recommendations. The analysis showed a positive association of adopting the Paleolithic diet in relation to weight loss. The effect was significant on weight, body mass index and waist circumference.

**Conclusion:**

The Paleolithic diet may assist in controlling weight and waist circumference and in the management of chronic diseases. However, more randomized clinical studies with larger populations and duration are necessary to prove health benefits.

**Trial registration:**

CRD42015027849.

## Introduction

Chronic noncommunicable diseases (CNCDs) are a matter of great concern to the world public health and the leading cause of death. In 2005, around 35 million deaths were attributed to such diseases, almost 60% of global mortality and 45.9% of the global burden of diseases [1]. If this trend is maintained, it is estimated that by 2020 CNCDs will account for 73% of deaths and 60% of disease burden [2].

According to the World Health Organization – WHO (2014) [1], CNCDs include cancer, diabetes, chronic respiratory disease, and cardiovascular diseases. According to the Institute of Medicine – IOM (2012) [3], CNCDs correspond to a larger group of diseases given the conditions grouped together (arthritis, cancer survivors, chronic pain, dementia, depression, type 2 diabetes mellitus, post traumatic disability conditions, schizophrenia and loss of sight and hearing) that characterize as chronic diseases those with long duration and limiting the routine activities of daily life.

Excess weight is associated with a significantly greater risk of developing chronic diseases and health problems that cause devastating consequences, in addition to increased mortality rates [4].

The onset of CNCDs has been related to factors such as smoking, alcohol consumption, low consumption of fruits and vegetables and high consumption of sodium and sugar [1]. Thus, diet plays an important role in the prevention and treatment of CNCDs, since it may have a positive influence, as a protective factor, or negative, as a risk factor, in the pathogenesis of these diseases [5].

Numerous diets are recommended in an attempt to combat CNCDs, whether in the preventive or control setting. Food and nutrition organizations in different countries set guidelines for healthy diets. In parallel, there is a growing popularity of “fad diets” that are spelled out in magazines and social media, especially appealing for quick results and with the weight loss approach. These diets generally have no scientific basis and can harm the health of those who adopt them [6].

In the field of evidence-based recommendations, there are valued food standards, regardless of country guidelines. One of the most accepted diets for the treatment and prevention of CNCD is the Mediterranean diet, which is characterized by a high intake of cereals, vegetables, fruits and olive oil; a moderate intake of fish and alcohol, mainly wine; and low intake of dairy products, meat and sweets [7]. The DASH diet (dietary approaches to stop hypertension), originally designed to control hypertension, is also mentioned. Lately, it has been advocated as a healthy eating pattern that helps to maintain weight, prevent heart disease and some cancers [8].

The Paleolithic diet has been gaining ground in the field of fad diets. It is based on food patterns of human Paleolithic ancestors, about 2.6 million to 10,000 years ago, a period that precedes the advent of industrial agriculture and is different from today’s modern society. In this period, the presence of hominins species (bipedal primates), a term previous to hominids, stood out. Food choices varied, since men were hunter-gatherers and often moved in search of food availability, which in turn varied according to geographic location and climate. However, there are universal characteristics of pre-agricultural hominin diets that are useful for understanding how the present Western diet may predispose modern populations to chronic diseases [9]. Although diets differed in the composition of macronutrients and in the proportion of food of animal and vegetable origin, there was a relatively common point in relation to exclusions, given the lack of some types of food in that period, such as dairy products, salt, alcohol, sugar, cereals and processed products [10, 11].

The Paleolithic diet has become so popular that it has led to the development of clinical trials evaluating its usefulness. It even deserved a recent systematic review with meta-analysis on its applicability in metabolic syndrome [12]. However, there have been conflicting results, some noting the positive effects of the Paleolithic diet on reducing the risk of diabetes, cancer, metabolic syndrome, cardiovascular diseases and type 2 diabetes [13, 14, 12], others mentioning deleterious effects [15], and others proving no effect [16].

The present study aims to analyze the influence of the Paleolithic diet with focusing on anthropometric (weight, body mass index and waist circumference) through a systematic review with meta-analysis.

## Methodology

The following question was used to perform the systematic review: What is the effect of the Paleolithic diet on anthropometric parameters (weight, body mass index and waist circumference) compared to other control diets based on recommendations in adults?

Only studies involving humans were included. The research included randomized studies on the use of the Paleolithic diet for prevention and control of CNCD, and published in Portuguese, English or Spanish. The definition of chronic diseases was in accordance with the IOM classification (2012) [3].

The PRISMA-P systematic review protocol was used [17, 18] and the study was registered in the PROSPERO platform under number CRD42015027849.

The search was performed until March 2019, and were found publications covering the period between 1969 and 2019. All studies found in the search were used, regardless of the date of publication.

The initial strategy for searching for descriptors or keywords included an initial search in MeSH (Medical Subject Headings), which has the controlled vocabulary for indexing articles used in PubMed. Then, the following descriptors were investigated, and based on the only systematic review performed with the Paleolithic diet by Manheimer et al. (2015) [7]:

- Related to the diet: paleolithic nutrition; palaeolithic nutrition; paleolithic diet; palaeolithic diet; paleolithic-type diet; palaeolithic-type diet; paleonutrition; palaeonutrition; paleo diet; palaeo diet; caveman diet; stone age diet; hunter gatherer diet; caveman cuisine; primal diet; evolution diet; primitive diet; ancestral human diet.
- Related to the type of study and subjects studied: randomized controlled trial; controlled clinical trial; randomized; placebo; drug therapy; randomly; trial; groups; humans.

Using the descriptors found, the following databases were searched: Latin American and Caribbean Literature in Health Sciences (LILACS), Public Medline (PubMed), Scientific Electronic Library Online (Scielo), Science Direct, Medical Literature Analysis and Retrieval System Online (Medline), Web of Science and SciVerse Scopus (Scopus). Boolean operators (OR or AND) were applied: OR was used to select articles containing any of the terms; AND was used to select only articles containing both descriptors [19].

Two authors independently reviewed the studies identified in the search according to inclusion and exclusion criteria.

The process of selection and cataloging of studies consisted of three stages called filters.

In the first screening, after reading of title and abstract, were selected articles on the theme that answered the two key questions: “Was the study conducted with humans?” and “Does the study evaluate the relationship of adopting the Paleolithic diet in the prevention and control of CNCD?”. When title and abstract were not enlightening, the article was searched in its entirety. This evaluation was done by two researchers independently. After this screening, each researcher presented a list of potential articles. The two lists were compared, and from the results was constructed a single list. In case of divergence, the article was evaluated by a third researcher.

The second filter was the stage of data extraction by using a form containing the following items: title, author, year of publication, city, type of study, beginning of study, objective, inclusion criterion, exclusion criterion, sex, age, diet, statistical analysis employed, results, and conclusion. Data was entered into the Excel program.

The third filter included a selection of publications based on quality criteria. For this, the GRADE (Grading of Recommendations Assessment, Development and Evaluation) system was used to evaluate the quality and strength of recommendations [20].

A statistical test of heterogeneity was performed for the selected studies. It was estimated by the Cochran Q test and I^2^ statistic. The heterogeneity was confirmed with a significance level of *p* ≤ 0.10. The I^2^ statistic describes the percentage of total variation of point estimates that can be attributed to the heterogeneity. For the I^2^ metric, were considered low, moderate and high values, and percentages of 25, 50 and 75%, respectively. Then, forest plot graphs were analyzed to examine the overall effect and evaluate the publication bias [21]. The summary measure was performed in Review Manager 5 (RevMan 5), and fixed effects were evaluated for weight, body mass index (BMI) and waist circumference (WC).

## Results

With the described research strategy were found 1224 articles. Based on exclusion criteria, the following were excluded: 12 articles because they were in another language (other than Portuguese, English and Spanish), five articles conducted on animals, and 136 articles not found in the databases.

The remaining 1088 articles were analyzed by two researchers based on the question ‘Can the Paleolithic diet help in the prevention and/or control of chronic diseases in humans?’. For this review, were used only articles that described anthropometric data.

**Fig. 1:**
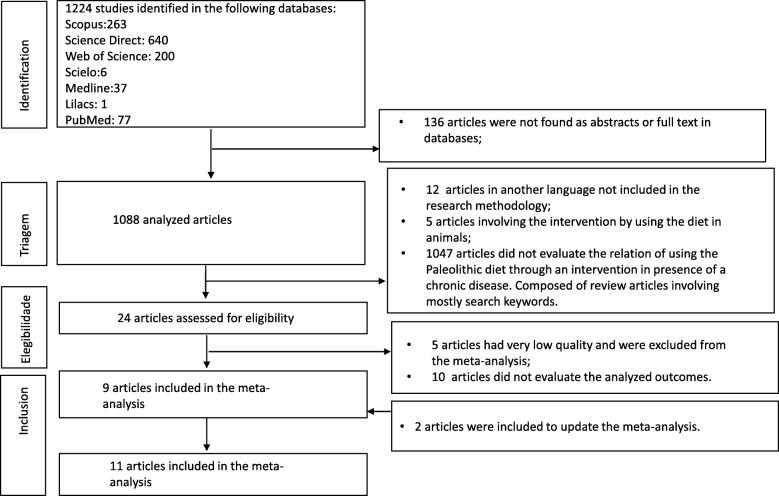
Details of the selection process. Studies identified after the 2019 search

We selected 31 articles without disagreement among researchers. However, one study was characterized as case-control and six were abstracts presented at scientific events. Therefore, the systematic review included 24 articles. After the quality analysis, nine articles were included because they presented quality ranging from two to three stars and involved the control of anthropometric markers as main or secondary outcomes (weight, body mass index - BMI and/or waist circumference - WC). 2 years after the review, data were updated and two more articles were included, as shown in Fig. 1.

Duration of interventions ranged from 2 weeks to 24 months, with participants assessed at baseline and after the intervention.

The Table 1 describes the articles that were included in the review. The quality analysis by using GRADE showed that in general, randomized intervention studies involving the Paleolithic diet have small samples, variations on the duration of interventions, and lack of information on the masking/blinding of the population involved. These aspects reduce the quality of the study, although there were no serious inconsistencies.

**Table 1:** Articles included in the review according to author, year of publication, type of study, characteristics of populations undergoing interventions, evaluated outcomes and analysis of quality

Variables Author/Year	Population characteristics	Participants Men/Women Paleolithic diet	Participants Men/Women Control diet	Mean age (years) Paleolithic diet	Mean age (years) Control diet	Duration (Months)	Evaluated outcomes	Secondary outcome evaluated	Quality of the study (GRADE)
Ahlgren et al., [22]	Overweight and obese women	0/6	0/6	64.5	70.6	24 months	Experiments on dietary change	Qualitative analysis	^b^
Otten et al., 2016 [23]	Obese postmenopausal women	0/25	0/16	61 ± 6	66 ± 2	24 months	Changes in liver fat and insulin sensitivity	Weight, BMI and WC	^c^
Pastore et al., 2015 [14]	Hypercholesterolemic patients	10/10	10/10	53 ± 7	53 ± 7	4 months	Dietary intake and plasma lipids	Weight	^c^
Masharani et al., 2015 [24]	Type 2 diabetes	14	10	58 ± 8	56 ± 13	3 months	Metabolic and physiological effects of diet	Biochemical markers	^b^
Bligh et al., 2015 [25]	Healthy population	24/0	–	27.9 ± 13.2	27.5 ± 12.68	6 months	Acute effects of meal on glycaemia, hormonal responses in the intestine and regulation of appetite	Hormones	^a^
Boraxbekk et al., 2015 [26]	Overweight and obese postmenopausal women	0/9	0/11	61.1 ± 1.6	61.6 ± 1.7	6 months	Improvement of episodic memory performance and change of associated functional brain responses	Weight, BMI and WC	^c^
Stomby et al., [27]	Overweight and obese women	0/27	0/22	–	–	24 months	Normalization of glucocorticoid metabolism	Weight, BMI and WC	^c^
Bisht et al., 2014 [28]	Multiple sclerosis patients	01/08	–	52.4 ± 4.1	–	12 months	Effect on perceived fatigue	Fatigue markers	^a^
Hanmarstrom et al., [29]	Overweight middle-aged women	0/8	–	57.5 ± 11	–	24 months	Barriers and facilitators for weight loss	Qualitative analysis	^a^
Boers et al., 2014 [30]	Metabolic syndrome patients	18	16	52 ± 10.2	55 ± 9	2 weeks	Alteration of metabolic syndrome characteristics	Weight, BMI and WC	^c^
Melberg et al., [31]	Obese postmenopausal women	0/35	0/35	59.9 ± 5.5	60.3 ± 5.9	24 months	Effect of the Paleolithic diet in obese women	Weight, BMI and WC only from the Paleolithic group	^a^
Frassetto et al., 2013 [32]	Type 2 diabetes patients	06/07	08/05	56 ± 12	56 ± 12	3 weeks	Effect on total acid production	BMI only from the Paleolithic group	^b^
Jonsson et al., 2013 [33]	Type 2 diabetes patients	10/03	–	–	–	3 months	Effect on satiety	Satiety markers	^a^
Ryberg et al., 2013 [34]	Obese menopausal women	0/10	0/10	–	–	1 month and 1 week	Effect on liver fat and insulin resistance	Weight, BMI and WC only of the Paleolithic group	^b^
Myhill e al., 2013 [35]	Myalgic encephalomyelitis patients	138	–	23.5 ± .5	–	–	Mitochondrial dysfunction	Markers of mitochondrial function	^a^
Jonsson et al., 2010 [36]	Coronary syndrome patients	14/0	15/0	–	–	12 weeks	Effect on satiety	Markers of satiety	^a^
Jonsson et al., 2009 [37]	Type 2 diabetes patients	01/06	04/02	66 ± 6	63 ± 6	6 months	Improvement of glycemic control in association with several cardiovascular risk factors	Weight, BMI and WC	^b^
Frassetto et al., 2009 [38]	Healthy population in physical activity	06/03	06/03	38 ± 12	38 ± 12	3 weeks	Effect on glycemic control and association with cardiovascular risk factors	BMI only from the Paleolithic group	^b^
Baumgartner et al., 2009 [39]	Healthy population in physical activity	05/05	–	25 ± 21	–	4 weeks	Effect on oral microbiota and clinical data	Present bacteria	^a^
Osterdahl et al., [16]	Healthy population	08/09	–	30 ± 10	–	3 weeks	Effect of the Paleolithic diet on the reduction of cardiovascular risk	Weight, BMI and WC	^a^
Lindeberg et al., 2007 [40]	Ischemic heart disease patients	14/0	15/0	65 ± 10	57 ± 7	12 weeks	Effect of glucose tolerance	Weight and WC	^c^
Smith et al., 2014 [15]	Healthy population	24/20	–	31.2 ± 0.3	–	2 months and 2 weeks	Effects on serum lipids	Weight	^a^
Genoni et al., 2016 [41]	Healthy women	0/22	0/17	47 ± 13	26.8 ± 7.2	1 months	Metabolic and cardiovascular effects	Weight and WC	^c^
Fontes-Vilalba et al., [42]	Type 2 diabetes patients	06/01	04/02	66 ± 6	63 ± 6	6 months	Effects on adipokines, glucagon, incretins and ghrelin	Weight	^b^
Blomquist C, et al. 2017 [43]	Healthy postmenopausal women	0/35	0/35	60 + 5.6	61 + 7	24 months	Android fat, weight, adipose gene expression, toll-like receptor 4, macrophage migration, Serum interleukin 6, tumor necrosis factor a levels and High-sensitivity C-reactive protein	Weight	^b^
Blomquist C, et al. 2018 [44]	Postmenopausal women with overweight	0/33	0/25	60 ± 5.5	62 ± 5.7	6 months	insulin sensitivity, decreased circulating triglycerides, gene expressions of CD36, fatty acid synthase and diglyceride acyltransferase 2	BMI and Body weight	^b^

*BMI* Body Mass Index

*WC* Waist Circumference

GRADE quality level [20]: ^a^very low; ^b^ low; ^c^ moderate

The analysis of the nine studies that analyzed anthropometric markers allowed the organization of data in three subgroups, namely variation of weight, BMI and WC.

The paleo and control diets and how to measure diet compliance were also evaluated, and Table 2 shows data from studies included in this review. In most studies, were used diets based on higher consumption of vegetables, whole grains and low-fat dairy for the control group. For the Paleo diet intervention group, diets were based on fish, lean meats, eggs, vegetables, fruits, and nuts, and exclusion of cereals, dairy products and sugar. The diets given to participants were evaluated through a food record of more than 2 days. Its composition was also described.

**Table 2:** Description of the Paleo diet, control diet and methodology of evaluation of diets

	Paleo diet	Control diet	Evaluation of dietary intake
Boers et al., 2014 [30]	Centesimal composition: not informed. Included: fish, lean meat, eggs, nuts, fruits, vegetables (dark green and cruciferous) and roots. Excluded: cereals, dairy products, legumes, salt, sugar and refined fat.	Centesimal composition: not informed. Included: according to Guidelines for a healthy diet of the Dutch Health Council.	Daily food record with telephone contact every other day.
Boraxbekk et al., 2015 [26]	Centesimal composition: Energy (Kcal):1.698 (235) Protein (%): 24 (2) Lipid (%): 44 (4) Carbohydrate (%): 28 (3) Included: fish, lean meat, eggs, nuts, fruits. Excluded: not informed.	Centesimal composition: Energy (Kcal): 1.676 (364) Protein (%): 19 (1) Lipid (%): 30 (6) Carbohydrate (%): 45 (6) Included: according to Nordic Nutrition Recommendations with low-fat dairy products and high-fiber products. Excluded: not informed.	Food record for 4 days, at the beginning and at 6 months of intervention.
Genoni et al., 2016 [41]	Centesimal composition: Energy (Kcal): 5915 (1452) Protein (%): 26.8 (7.2) Lipid (%): 39.8 (9.6) Carbohydrate (%): 27.8 (8.05) Included: fish, lean meat, eggs, nuts, fruits and vegetables. Excluded: cereals, grains, dairy products, legumes and potatoes.	Centesimal composition: Energy (Kcal): 6657 (1725) Protein (%): 21.7 (5.6) Lipid (%): 32.6 (7.3) Carbohydrate (%): 40.6 (9.4) Included: five food groups according to Australian dietary recommendations; higher intake of vegetables, fruits and whole products; consumption of low-fat dairy products; Reduced foods: fat, cakes, cookies, sugary drinks and sweets.	Food record for 3 days (2 days per week and one weekend day) before starting the intervention and at days 26–28 after the intervention.
Jonsson et al., 2009 [37]	Centesimal composition: Energy (Kcal):1445 (367) Protein (%): 24 (3) Lipid (%): 39 (5) Carbohydrate (%): 32 (7) Included: fish, lean meat, eggs, nuts, fruits, vegetables (dark green, cruciferous), roots, canola oil, olive oil and wine. Excluded: grains, bakery products, dairy products, legumes, salt, sugar, sugary drinks, refined fat, and beer.	Centesimal composition: Energy (Kcal):1456 (312) Protein (%): 20 (4) Lipid (%): 34 (6) Carbohydrate (%): 42 (7) Included: fruits, vegetables, whole bread and other whole grains, and legumes. Reduced foods: total fat and salt.	Food record for four consecutive days (including a weekend day), beginning sex weeks after the start of intervention.
Lindeberg et al., 2007 [40]	Centesimal composition: Energy (Kcal): 1344 (521) Protein (%):27.9 (6.8) Lipid (%):26.9 (6.4) Carbohydrate (%):40.2 (8.3) Included: fish, lean meat, eggs, nuts, fruits and vegetables. Excluded: grains, dairy products, salt, sugar and refined fat.	Centesimal composition: Energy (Kcal):1795 (306) Protein (%): 20.5 (3.6) Lipid (%): 24.7 (4.3) Carbohydrate (%):51.7 (5.3) Included: according to the Mediterranean Diet, based on fish, whole grains, low-fat dairy products, potatoes, vegetables, fruits, and refined fats, rich in monounsaturated fatty acids and alpha-linolenic acid.	Food registration during four consecutive days (including a weekend day), beginning 15 ± 5 days after the start of intervention.
Otten et al., 2016 [23]	Centesimal composition: not informed. Included: fish, seafood, lean meat, eggs, nuts, fruits and vegetables Excluded: cereals, dairy products, legumes, salt and sugar.	Centesimal composition: not informed. Included: according to Nordic Nutrition Recommendations with increased intake of fish, fruits, vegetables, whole grains, meat. Dairy products should be low in fat.	Food records during 4 days (3 days a week and one weekend) performed two to 4 days after the start of intervention, monthly up to 6 months and then, at nine, 12, 18 and 24 months.
Pastore et al., 2015 [14]	Centesimal composition: Women: Energy (Kcal)**:** women 1719.7 (262.7); men 2101.8 (382.2) Protein (%): 37 Lipid (%):40 Carbohydrate (%): 23 Included: lean meat, eggs, nuts, fruits and vegetables. Excluded: grains, dairy products and legumes.	Centesimal composition: Women: Energy (Kcal): 2269 (310.5) Protein (%): 17 Lipid (%): 23 Carbohydrate (%): 60 Men: Energy (Kcal): 2866.1 (262.7) Protein (%):21 Lipid (%):23 Carbohydrate (%):56 Included: diet rich in fruits and vegetables, whole grains with little or no salt and fish at least twice a week. Reduced food: sugary foods and drinks.	From a series of ten 24-h reminders recorded at the beginning and during each phase of the diet.
Fontes-Vilalba et al., [42]	Centesimal composition: not informed. Included: lean meat, fish, fruits, vegetables (leafy and cruciferous), eggs and nuts. Excluded: dairy, grains, legumes, refined fats, sugar, sweets, soft drinks, beer and salt.	Centesimal composition: not informed. Included: higher intake of vegetables, fruits, roots, whole bread and other whole grains and legumes. Reduced food: total fat, and more unsaturated fat and salt.	Not informed.
Stomby et al., [27]	Centesimal composition: not informed. Included: fish, lean meat, eggs, nuts, fruits, vegetables and roots. Excluded: not informed.	Centesimal composition: not informed. Included: according to Nordic Nutrition Recommendations, low-fat dairy products and high-fiber products.	Food registration during 4 days (3 days a week and one weekend) performed two to 4 days after the start of intervention, monthly up to 6 months and then at nine, 12, 18 and 24 months.
Blomquist C, et al. 2017 [43]	Centesimal composition: not informed. Included: lean meat, fish, eggs, vegetables, fruits, red fruits, nuts, avocados and oils. Excluded: dairy products, cereals, added salt, refined fats and sugar.	Centesimal composition: not informed. Included: according to Nordic Nutrition Recommendations with meat, fish, vegetables, fruits and low-fat dairy products. Excluded: not informed.	Estimated food records during 4 days at baseline, 6 months and 24 months.
Blomquist C, et al. 2018 [44]	Centesimal composition: Not informed. Included: lean meat, fish, eggs, vegetables, fruits, nuts, avocado, rapeseed oil and olive oil. Excluded: dairy, cereal, salt, refined fat and refined sugar.	Centesimal composition: Not informed. Included: according to Nordic Nutrition Recommendations with meat, fish, vegetables, fruits and low-fat dairy products. Excluded: not informed.	Not informed.

The three markers were evaluated in five studies [23, 26, 27, 30, 37]; weight and WC were evaluated in three studies [40, 41, 44], and weight only was evaluated in three other studies [14, 42, 43].

Among the articles in which was performed the meta-analysis, five (45,5%) involved women mostly in the menopausal or postmenopausal period, and was evaluated the effect of diet on weight loss [23, 26, 27, 43, 44]. There was an article on healthy women with a focus on disease prevention [41]. As for the others, two (18,2%) clinical trials were performed with subjects with type 2 diabetes [37, 42], one (9,1%) in heart disease patients [40], one (9,1%) in patients with metabolic syndrome [30], and one (9,1%) in hypercholesterolemic individuals [14].

**Fig. 2:**
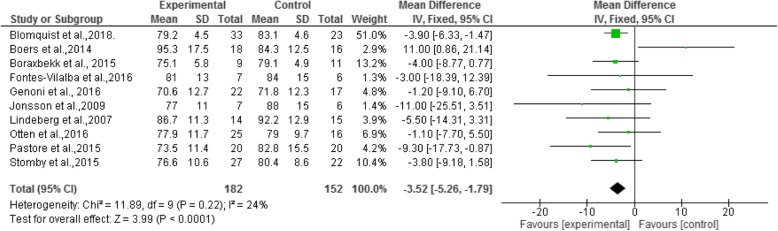
Forest plot - Mean body weight differences of a population participating in a random clinical trial using the Paleolithic diet

By analyzing the body weight, was found a decrease in the mean weight (− 3.52 kg), and the Confidence Interval (95%) ranged from − 5.26 to − 1.79. The forest plot (Fig. 2) shows a relationship between weight loss and the adoption of the Paleolithic diet (I^2^ = 24% and *p* = 0.22).

**Fig. 3:**
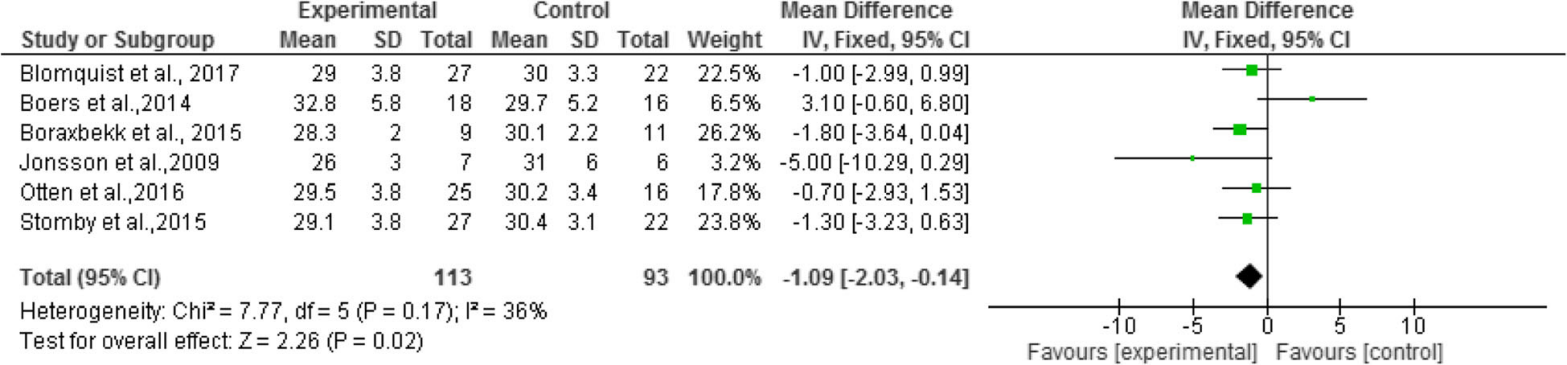
Forest plot - Mean differences of Body Mass Index (BMI) of a population participating in a randomized clinical trial using the Paleolithic diet

**Fig. 4:**
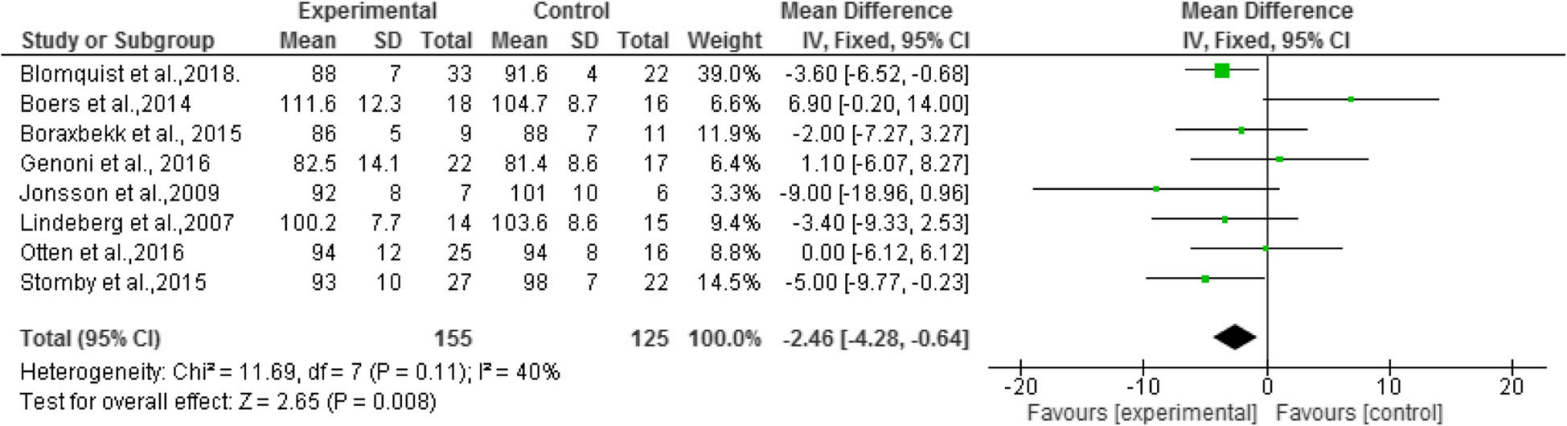
Forest plot - Mean differences of Waist Circumference (WC) of a population participating in a randomized clinical trial using the Paleolithic diet

These effects were also found in BMI, with a mean reduction of − 1.09 kg/m^2^, Confidence Interval (95%) from − 2.03 to − 0.14. The forest plot (Fig. 3) shows no relation between BMI and the Paleolithic diet (I^2^ = 36% and *p* = 0.17). For WC, the mean reduction was − 2.46 cm, and for the confidence interval (95%) from − 4.28 to − 0.64, demonstrating a relationship between WC and the adoption of the Paleolithic diet (I^2^ = 40% e *p* = 0.11), as shown in the forest plot, Fig. 4.

## Discussion

The present study evaluated randomized clinical trials (RCT), which is considered a gold standard design because it is the least affected by the influence of confounding factors and biases [45].

Regardless of the RCT design, some characteristics must be observed in order to extrapolate the results of a study to a certain clinical reality. The closer the characteristics considered by the study, the more relevant the evidence becomes.

Four basic aspects specifically determine validity, namely: participants, interventions, environment, and outcomes. With respect to participants, it is important that characteristics are close to those of the population of interest. Aspects such as age, sex, severity of disease, risk factors and associated comorbidities should be carefully considered [35]. In controlled studies, the control group and the intervention group were similar regarding age, sex and health condition. The I^2^ metrics of the eleven studies included was 24, 36 and 40% for weight, BMI and WC, respectively, showing homogeneity mainly in relation to weight.

When comparing the eleven studies, the population groups differed significantly in clinical characteristics, since individuals were diabetic, hypercholesterolemic, obese, healthy and with metabolic syndrome.

The size of samples evaluated in the included studies could be considered small, as the largest intervention had 41 participants. Larger samples allow better identification of relevant effects from the clinical and public health point of view, albeit of small magnitude [23].

Interventions ranged from 2 weeks to 24 months. The analyzed studies indicate a relation between the positive results of the Paleolithic diet and the short duration of the intervention, and its beneficial effects on metabolism are reduced as the intervention continues. In the studies, the sixth month appeared as the time to obtain better results, as also observed by Otten et al. (2016) [23] and Stomby et al. (2015) [27].

The eleven studies analyzed the body weight variation throughout the intervention of Paleolithic diet, and better results were found among overweight and obese women [26, 27]. The study by Otten et al. (2016) [23] also had the same population profile but found lower weight loss.

A fact limiting the verification of effects is the initial difference in the analyzed variable when comparing the two types of diets, as in the study by Boers et al. (2014) [30], in which the group adopting an evidence-supported diet had a lower initial mean weight than the group adopting the Paleolithic diet. This study, in particular, also had a very short duration (2 weeks), and this may have influenced the findings.

The BMI outcome was evaluated in six studies and was associated with the adoption of the Paleolithic diet (*p* = 0.02). The study with the best findings in relation to this outcome was that of Boraxbekk et al. (2015) [26], which involved overweight and obese women and lasted for 6 months. Of the six studies involving BMI, all showed a significant (*p* < 0.05) reduction of this anthropometric marker when analyzed alone, without comparison to the group that adopted the other diet.

In eight studies, waist circumference was associated with the adoption of the Paleolithic diet (*p* = 0.008). In the study by Boraxbekk et al. (2015) [26], this measurement decreased, which is in line with results of weight reduction and improvement of BMI. The effect found in the study by Otten et al. (2016) [23] was different, and indicated weight reduction and increased WC. This marker was evaluated by Manheimer et al., 2015 [12], who found a significant reduction in waist circumference (I^2^ = 52%). In this outcome, the highest weight was from the study by Blomquist et al., 2018 [43].

The results of this study were significant for weight loss, BMI and waist circumference. A possible hypothesis to explain the effect of the Paleolithic diet on weight loss, is its satietogenic effect, as verified by Bligh et al., 2015 [38], who tested the acute effect of meals based on the Paleolithic diet on biochemical markers of satiety compared to a guideline-based diet [1]. Twenty-four men aged between 18 and 60 years old were healthy and had BMI between 18 and 27 Kg/m^2^. Concentrations of glucagon-1 (GLP-1) and Peptide YY (PYY) peptides were significantly increased over 180 min with the use of different formulations of the Paleolithic diet compared to the control diet.

This meta-analysis was focused on anthropometric markers by evidencing favorable effects of adopting the Paleolithic diet to body weight BMI and waist circunference. The study adds to the review by Manheimer et al. (2015), in which was found a significant improvement in some markers of metabolic syndrome when adopting this diet.

For future reviews, a greater number of quality clinical trials is required for better definitions. A longer follow-up and larger sample size are recommended in future clinical trials on the subject, besides a greater standardization of the Paleolithic diet used. Studies presenting the biochemical results and proven pathophysiological mechanisms of this diet are also scarce.

## Conclusion

The adoption of the Paleolithic diet is associated with weight loss, BMI and WC. Still, this diet can influence the prevention and control of chronic diseases, since excess weight is a risk factor for their development.

In addition to any food fads, the present study points to the need for further research to evaluate other effects in controlled and well-designed studies regarding sociodemographic, economic, cultural and clinical characteristics of the selected sample, time of adoption of the diet, and type of Paleolithic diet used.

## Data Availability

Data sets used and / or archived during the occurrence cycle are available without reasonable collection.

## Abbreviations

BMI: Body mass index
CNCD: Chronic noncommunicable diseases
DASH: Dietary approaches to stop hypertension
GLP-1: Glucagon-1
GRADE: Grading of Recommendations Assessment, Development and Evaluation
IOM: Institute of Medicine
LILACS: Latin American and Caribbean Literature in Health Sciences
Medline: Medical Literature Analysis and Retrieval System Online
MeSH: Medical Subject Headings
PubMed: Public Medline
PYY: Peptide YY
RCT: Randomized clinical trials
Scielo: Scientific Electronic Library Online
WC: Waist circumference
WHO: World Health Organization
